# A systematic review of parent based programs to prevent or reduce alcohol consumption in adolescents

**DOI:** 10.1186/s12889-019-7733-x

**Published:** 2019-11-04

**Authors:** Erin Hurley, Timo Dietrich, Sharyn Rundle-Thiele

**Affiliations:** Social Marketing @ Griffith, Griffith University, 170 Kessels Road, Nathan, Qld 4111 Australia

**Keywords:** Parent, Review, Alcohol, Prevention, Schools

## Abstract

**Background:**

Adolescent alcohol consumption is an issue of ongoing concern and programs targeting parents have been identified as an important component in minimizing and preventing alcohol related harm in adolescents. This paper aims to evaluate existing parent based alcohol education programs with a focus on understanding parent specific outcomes including parental attitudes, parent-child communication, alcohol specific rule setting and parental monitoring; study quality, the extent of stakeholder engagement in program design and the level of theory application.

**Method:**

A systematic review of electronic databases EBSCO, Emerald, ProQuest, PubMed, Ovid, ScienceDirect, Taylor and Francis and Web of Science was conducted from database inception to August 2019. A total of 4288 unique records were retrieved from the eight databases. Studies were included if they evaluated school based alcohol education programs that included a parent component and detailed outcome measures associated with parent data. The methodological quality of the included studies was assessed using the Effective Public Health Practice Project (EPHPP) quality assessment tool.

**Results:**

In total 17 studies qualified for assessment, detailing 13 individual parent programs. Of these, ten programs demonstrated positive effects in at least one parent reported outcome measure. Stakeholder engagement during the design of programs was lacking with the majority of programs. One third of the programs did not report theory use and when theory was used reporting was weak with three programs applying theory, five testing theory and none building theory. According to the EPHPP tool, overall ten programs were rated as weak, three as moderate and none as strong.

**Conclusion:**

Future studies are recommended to further enhance the effectiveness of parental programs by improving study quality, increasing stakeholder engagement and increasing the level of theory application and reporting.

## Introduction

Parents remain one of the most important social influencers in preventing and reducing adolescents’ alcohol consumption [1]. Several studies indicate a positive association between specific parenting factors and adolescents’ alcohol use [2–4]. Research shows that adolescents’ whose parents have restrictive attitudes regarding underage drinking are less likely to engage in risky drinking behaviors [5, 6]. Furthermore, high quality parent-child communication [7], including the communication of strict alcohol specific rules [8, 9], and parent’s monitoring of adolescent’s activities and whereabouts [4, 10] are associated with reduced levels of alcohol consumption among adolescents. Therefore, parents are key stakeholders in alcohol prevention strategies and alcohol-specific programs targeting parents remain an important component of multi-faceted approaches to minimizing alcohol-related risks in adolescents [1, 11, 12].

While evidence of the protective role that parents can play in delaying or reducing the amount of alcohol consumed by adolescents, and effectiveness of parent alcohol programs exists [1, 13], less is known about the effectiveness of programs from a parent’s perspective, stakeholder engagement during program design and theory utilization. A recent meta-analytic study identified evidence of parent alcohol programs efficacy in preventing or reducing alcohol use [14]. Other systematic reviews have examined the efficacy of parent alcohol programs on preventing alcohol misuse in adolescents (see for example [1, 13, 15, 16]). For example, Newton et al., [1] found that nine out of ten combined student and parent alcohol programs showed effectiveness in delaying or reducing alcohol and drug use in adolescents. While these reviews advance understanding of the effect parent alcohol programs delivered within multi-component settings have on adolescents, they do not indicate the impact on parents who participate in programs, thereby limiting insights into how effectiveness for parents may be enhanced.

The Kuntsche et al., [17] review focused attention on the efficacy of parent alcohol programs on parenting specific factors. Their findings indicated desirable effects of parent factors such as rule-setting, monitoring and parent–child communication. However, the reported outcome measures were based on adolescent self-reports rather than parental responses directly. A systematic review of studies focused on understanding program effects for parents themselves does not exist. This limits understanding given there may be discrepancies between parent and adolescent reports of parenting behaviors [18, 19]. Extending understanding of program effectiveness from a parent perspective allows for a more comprehensive understanding to emerge.

Inclusion of multiple stakeholders across the span of a program can improve behaviour change outcomes [20], through enhanced acceptance and adoption of programs into the community [21]. Stakeholder engagement can occur from early formative research and concept development stages [22], through to program implementation and evaluation stages [23, 24]. Freeman [25] defines stakeholders as “any group or individual who can affect or is affected by the achievement of an organization’s objective” (p.53). This involves the meaningful engagement of individuals or groups who are either affected by program implementation or have the power to affect the outcome of the program (e.g. government, local communities, target user groups, health care providers) [26]. For example, stakeholder involvement may involve collaborating with target users and key community members during the formative research process to generate insights to guide program development [22].

However, stakeholder engagement is often limited to single stakeholder perspectives [27] and stakeholder participation in program design is often overlooked, limiting program potential [28]. Understanding and providing value for multiple stakeholders can be important indicators of change [20] and may result in better outcomes [29]. Furthermore, processes which empower stakeholders during early design stages may improve program success through the consideration and integration of stakeholder insights in core program elements [30], maximizing stakeholder support [28]. Importantly, the potential for stakeholders to influence program outcomes may be greater during initial program development stages when they are provided more freedom to shape program goals and outcomes [31]. However, current alcohol programs lack the inclusion of stakeholder input during program design [28, 32], failing to acknowledge new information, ideas and stakeholder perspectives that are more likely to improve program design [29].

Stakeholder engagement can occur in different forms from less involved methods whereby stakeholders have no power in the decision making process [33], to more collaborative methods that at the highest level strive for stakeholder empowerment [34]. Empowerment is characterized by an organizations willingness and capacity to share power with key stakeholders [34]. Empowering stakeholders during program design stages may; 1) allow for conflicts to be resolved before they arise during program design, implementation and evaluation [35], 2) lead to greater program innovation [36], and 3) improve program support resulting in a greater chance for sustainable change [37]. Given the above benefits of stakeholder engagement during program design this systematic review evaluated the level of stakeholder engagement during the design stage of parent alcohol programs.

Theories can be used in the development of programs to effect better outcome change [38], through influencing constructs that are known to cause the specific behavior [39]. French et al., [40] state that an appropriate theory should be identified to inform and guide program development, implementation and evaluation. The application of behaviour change theories provides a greater understanding of the mechanisms leading to change [41] and allows for the identification and selection of appropriate behaviour change techniques [38].

Parent programs are designed to effect change in parenting behaviours associated with underage drinking. Ecological theories of behaviour change such as ecodevelopmental theory and social cognitive theory (SCT) extend focus beyond individual factors, emphasizing social and environmental contexts [42, 43]. Such theories suggest that adolescents’ social and environmental influences including parents, schools and communities have a profound impact on adolescent problem behaviours such as underage drinking. For example, ecological theory is focused on targeting specific contextual risk (e.g. parental supply of alcohol) and protective factors (e.g. parental monitoring), to facilitate positive adolescent development [44]. In a review of parent programs for adolescent substance use and problem behaviours Ladis et al., [45] identified family systems theory and ecological theory as the guiding frameworks used in the majority of identified programs. In line with ecological theories of behaviour change, parent attitudes and behaviours play an important role in influencing adolescent alcohol use and parental attitudes and behaviours have thus been identified as relevant in the design, implementation and evaluation of parent alcohol programs.

However, many programs are not utilizing theory [46–48] and when theory use is reported the level of theory utilization remains low [49, 50]. Moreover, mixed construct and measure use is observed further limiting scientific advancement [51]. Without the detailed reporting of constructs and application of consistent measures in parent programs, attempts to synthesize cannot be undertaken. The systematic application of theory extends evidence by allowing the replication of practices across a range of contexts [51]. With theory use offering the potential to further extend program outcomes [52], this review aims to examine the extent of theory use in parent alcohol programs.

Taken together, while evidence indicates that program design should incorporate stakeholder engagement [53] and be theoretically guided [38], available reviews do not provide guidance on the extent of stakeholder engagement and theory use. The aims of this systematic review study are threefold. First, this study aims to understand outcomes experienced for parents participating in parent alcohol programs. Second, it aims to identify the extent of stakeholder engagement in program design. Finally, this review examines the extent of theory utilization to advance understanding of theory use in program design.

## Method

### Search strategy

Peer-reviewed literature was systematically searched to identify relevant studies published between database inception and August 2019 from eight databases including; PubMed, EBSCO, Emerald, ProQuest, Ovid, ScienceDirect, Taylor & Francis and Web of Science. A copy of the review protocol was not prospectively registered, however, the systematic search of the literature followed the Preferred Reporting Items for Systematic Reviews and Meta-Analyses (PRISMA) guidelines [54]. Due to the heterogeneity of the identified programs in regards to study populations and outcome measures, meta-analysis was not possible [55, 56]. Eight databases were searched using the keywords alcohol*, parent* and school* in combination with *randomized controlled trial*, *intervention*, *program*, and *evaluation*. See Table 1 for a detailed list of search terms.

**Table 1:** Search terms used to search for articles in eight databases

Search terms used to search for articles	
PubMed	(((alcohol) AND (intervention* OR Randomi?ed. Controlled Trial OR evaluation OR trial OR program*)) AND parent*) AND school*
EBSCO	AB alcohol AND AB (intervention* OR Randomi?ed. Controlled Trial OR evaluation OR trial OR program*) AND AB parent* AND AB school* TI alcohol AND TI (intervention* OR Randomi?ed. Controlled Trial OR evaluation OR trial OR program*) AND TI parent* AND TI school* SU alcohol AND SU (intervention* OR Randomi?ed. Controlled Trial OR evaluation OR trial OR program*) AND SU parent* AND SU school*
Emerald	[Abstract: alcohol] AND [[Abstract: intervention*] OR [Abstract: Randomi?ed. Controlled Trial] OR [Abstract: evaluation] OR [Abstract: trial] OR [Abstract: program*]] AND [Abstract: parent*] AND [Abstract: school*] [Publication Title: alcohol] AND [[Publication Title: intervention*] OR [Publication Title: Randomi?ed. Controlled Trial] OR [Publication Title: evaluation] OR [Publication Title: trial] OR [Publication Title: program*]] AND [Publication Title: parent*] AND [Publication Title: school*] [Keywords: alcohol] AND [[Keywords: intervention*] OR [Keywords: Randomi?ed. Controlled Trial] OR [Keywords: evaluation] OR [Keywords: trial] OR [Keywords: program*]] AND [Keywords: parent*] AND [Keywords: school*]
ProQuest	ab(alcohol) AND ab(intervention* OR Randomi?ed. Controlled Trial OR evaluation OR trial OR program*) AND ab(parent*) AND ab(school*) ti(alcohol) AND ti(intervention* OR Randomi?ed. Controlled Trial OR evaluation OR trial OR program*) AND ti(parent*) AND ti(school*) mainsubject(alcohol) AND mainsubject(intervention* OR Randomi?ed. Controlled Trial OR evaluation OR trial OR program*) AND mainsubject(parent*) AND mainsubject(school*)
Ovid	(alcohol and (intervention* or Randomi?ed. Controlled Trial or evaluation or trial or program*) and parent* and school*).ab (alcohol and (intervention* or Randomi?ed. Controlled Trial or evaluation or trial or program*) and parent* and school*).kf (alcohol and (intervention* or Randomi?ed. Controlled Trial or evaluation or trial or program*) and parent* and school*).ts (alcohol and (intervention* or Randomi?ed. Controlled Trial or evaluation or trial or program*) and parent* and school*).at (alcohol and (intervention* or Randomi?ed. Controlled Trial or evaluation or trial or program*) and parent* and school*).sh.
ScienceDirect	TITLE-ABSTR-KEY(alcohol) and TITLE-ABSTR-KEY(intervention* OR Randomi?ed. Controlled Trial OR evaluation OR trial OR program*) and TITLE-ABSTR-KEY(parent*) and TITLE-ABSTR-KEY(school*)
Taylor & Francis	[All: alcohol] AND [[All: intervention*] OR [All: randomi?ed]] AND [All: controlled] AND [[All: trial] OR [All: evaluation] OR [All: trial] OR [All: program*]] AND [All: parent*] AND [All: school*]
Web of Science	TOPIC: (alcohol)*AND* TOPIC: (intervention* OR Randomi?ed. Controlled Trial OR evaluation OR trial OR program*) *AND*TOPIC: (parent*) *AND* TOPIC:(school*) TITLE: (alcohol)*AND* TITLE: (intervention* OR Randomi?ed. Controlled Trial OR evaluation OR trial OR program*) *AND*TITLE: (parent*) *AND* TITLE: (school*)

A total of 6837 records were retrieved as summarized in Table 2. Each record was downloaded to Endnote and 2549 duplicates were removed. Inclusion and exclusion criteria were applied to the remaining 4288 unique records to ensure they were accurate representations of studies evaluating parent alcohol programs.

**Table 2:** Databases and records reviewed in initial search

Database	Number of records retrieved
EBSCO All Databases	530
Emerald	53
ProQuest All Databases	956
PubMed	1552
Ovid All Databases	1933
ScienceDirect	138
Taylor & Francis	145
Web of Science	1530
Total	6837

### Inclusion and exclusion criteria

Studies published in English and meeting the following criteria were included in the review: 1) detailed parent reported outcome measures on any factors related to general parenting or substance-related parenting, 2) published between database inception and August 2019, 3) reported results of an alcohol prevention program that involved an element of parent training, 4) included a control or comparison group, and 5) included parents of adolescents’ aged 10–18 years old as substance use typically first occurs in adolescence.

**Fig. 1:**
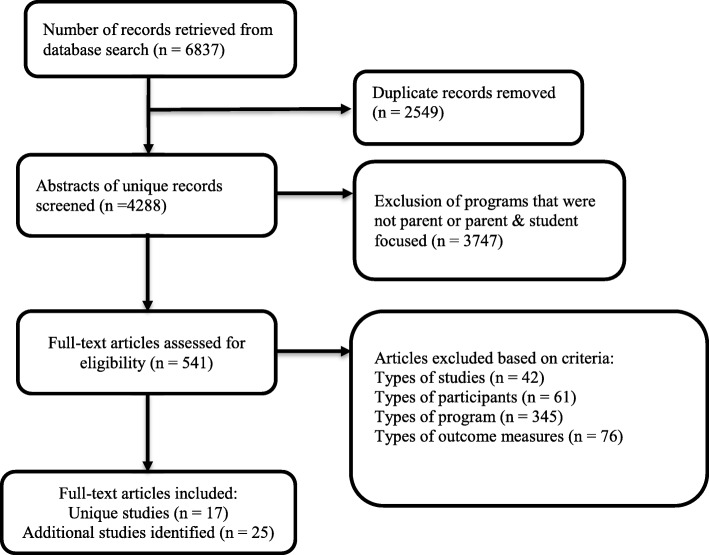
PRISMA diagram showing systematic search process

Studies were excluded if; 1) they did not evaluate a universal program (i.e. studies targeting specific populations such as immigrant families), 2) there was no program implemented, 3) they did not assess parent reported outcome measures associated with general or alcohol specific parenting behaviours and, 4) they were not a journal article. The flowchart based on the PRISMA guidelines [54] is shown in Fig. 1. In total 17 studies detailing 13 individual parent programs were identified prior to forward and backwards searching. To allow for accurate reporting of program development, implementation and evaluation, forward and backward searching was undertaken. Specifically, searches using authors’ names and program names were undertaken in Google Scholar. A further 25 relevant studies detailing further information on the included programs were located. In total 42 studies were included in the analysis of 13 different programs. The full list of the 42 studies for the 13 programs can be found in Table 4 in Appendix.

### Article screening

Two independent reviewers screened titles, abstracts, and full texts of potential articles. A third reviewer resolved disagreements regarding inclusion of a study. Following the application of the exclusion criteria, 17 studies evaluating 13 universal parent alcohol programs on parent outcome measures were identified.

### Data extraction and analysis

The included studies were analyzed in terms of; 1) program effectiveness on parent reported outcome measures, 2) the level of stakeholder engagement in program design, 3) the level of theory use and, 4) the quality of program design and delivery.

#### Parent outcome measures

Data associated with general or alcohol specific parenting behaviours as reported by parents were extracted from the included studies. Key outcome measures included parents’ attitudes towards underage drinking, parent-child alcohol specific communications, alcohol specific rule setting and parental monitoring. These factors have been identified as important influencers of adolescent alcohol consumption [17, 57].

#### Level of stakeholder engagement

The framework used to assess the level of stakeholder engagement has been used in previous studies [30, 58] and consisted of five levels: 1) Inform, which refers to one way communications with stakeholders to inform or educate; 2) Consult, which refers to gaining feedback and information from stakeholders in limited two-way communications; 3) Involve, which refers to working directly with stakeholders in multi-way communications to ensure concerns are understood and considered; 4) Collaborate which refers to partnering with stakeholders to develop joint plans of action; and 5) Empower which refers to enabling stakeholders to make the final decisions [59].

#### Level of theory utilization

Reported theory use and the extent of theory utilization was extracted and analyzed from the included studies as it has been linked to improved program outcomes [60]. The framework used to assess the level of theory utilization has been used in previous systematic reviews [49, 61] and is comprised of four levels, namely 1) informed by theory, whereby the study explicitly mentions theory but fails to apply a theoretical framework in study components or measures, 2) applied theory, whereby several theoretical constructs are applied to the study, 3) testing theory whereby at least half of the theoretical constructs are explicitly measured, and 4) building theory, whereby theory is revisited or created [49].

#### Quality assessment

The quality of the included studies was assessed using the Effective Public Health Practice Project (EPHPP) quality assessment tool for quantitative studies [62]. The EPHPP tool is suitable for evaluating multiple program study designs [63] and has been used to assess the quality of school-based programs in previous reviews [49, 64, 65]. The assessment tool has been validated [66, 67] and is suitable for use in systematic reviews of effectiveness [63]. The EPHPP tool rates each study according to six program aspects including selection bias, study design, control of confounders, blinding, data collection methods, and withdrawal and drop-out rates [67]. Each individual aspect is rated weak, moderate or strong and an overall rating is applied to each study [67]. All studies assessed through the EHPHH tool were rated by at least two researchers and inter-reliability scores exceeded the > 80% threshold. Discrepancies were discussed and resolved with all authors.

## Results

### Description of included studies

In total, 13 unique programs reported in 42 studies qualified for inclusion. Two programs were culturally adapted. Project Northland was culturally adapted for Russia [55], and Orebro (later named Effekt) was culturally adapted for Estonia [68]. The Orebro program was trialed on two separate occasions in Sweden [69, 70]. The majority of programs identified were conducted in the USA (46%, *n* = 6). The sample size of parents ranged from 64 [71] to 2048 [72]. Parent based programs are typically delivered through means of; workshops [73–75]; posting materials via mail [69, 72]; and take home materials from school [55, 76, 77]. The majority of programs focused on reducing parents permissive norms towards underage drinking [68, 69, 72–74]; encouraged parents to set clear alcohol specific rules [71, 72, 74, 75, 77] and aimed to increase parent-child communication [55, 72, 74, 78]. The follow-up assessment period ranged from immediately post program [79] to 36-months [69]. Standard health education was administered to the majority of the control conditions in each trial, however, informational booklets on different aspects of adolescent development were mailed to control parents in the Preparing for the Drug Free Years (PDFY) trial [75]. An additional file provides an overview of the characteristics of the studies included in the review (see Additional file 1).

### Efficacy of included studies on parent outcome measures

#### Parental restrictive attitudes towards underage drinking

Parents’ restrictive attitudes towards underage alcohol use was the most commonly measured parenting behavior and significant effects were observed in seven of the nine programs. Parents participating in the Orebro program had more restrictive attitudes towards underage drinking from baseline to 12, 30 [70] and 36 months [69]. Pettersson et al., [73] found that parents participating in the Strong and Clear program maintained their restrictive attitudes, while parents in the control group adopted more lenient attitudes towards adolescent drinking over time with a small to moderate program effect size reported. Furthermore, parents participating in both the Project Northland program [72] and the Prevention of Alcohol use in Students (PAS) program [78] had significantly more restrictive attitudes concerning the degree to which they found it acceptable for adolescents to drink in various situations. While the Youth and Alcohol program reported no program effect on parental attitudes towards alcohol over time, it is important to note that both the program and control groups had quite strict attitudes at baseline and a further increase in the scores was not anticipated [74].

#### Parent-child communication

Of the six programs measuring parent-child communication [71, 74, 76, 77, 79, 80], five demonstrated significant program effects. For example, Beatty et al., [76] found that intervention group parents were more likely to have a conversation regarding alcohol with their adolescents than those in the control. In addition, the authors found that the conversations were more likely to have occurred recently, included discussion about a larger variety of topics and lasted for a longer duration than control group parents. Furthermore, parents participating in the both the Families in Action and Family Matters program reported having significantly more parent-child discussions regarding the influence of peers and the media on their adolescents’ alcohol consumption [77, 79]. In contrast, Ennett et al., [77] measured communication using a single item that assessed the extent parents provided their adolescents with explanations when requesting them to do something, and found no difference between intervention and control groups at 3 month follow-up.

#### Alcohol specific rule setting

A significant effect was observed in four of the five programs measuring parents’ restrictive rules concerning their adolescents’ alcohol use [77, 78, 81, 82]. For example, parents who participated in the PAS program reported an increase in the degree of alcohol specific rule setting (e.g. allowing adolescents to drink at home, allowing adolescents to drink at a party with friends) from baseline to 10 months [78] and 34 months follow-up [81]. Similarly, both the Family Matters and PDFY programs observed a significant increase in parents’ restrictive rules regarding their adolescent alcohol consumption [77, 82].

#### Parental monitoring

Across the five programs measuring parental monitoring practices no improvements were observed [72, 74, 77, 80, 82]. This lack of effect was observed in both short term and long term follow ups. For example, across various programs participants demonstrated no increase in parental monitoring immediately post program [83], and at 4 weeks [80], 8 weeks [82], and 24 months [72] post program completion. Furthermore, parents participating in the Youth and Alcohol program in Norway reported no increase in their knowledge of their adolescents’ leisure time activities over a 28 month period (baseline to four, six and 28 months) [74]. However, at baseline parents in both the intervention and control groups reported an already high level of knowledge of their adolescents’ leisure time activities [74]. Finally, Ennett et al., [77] saw no significant increase in intervention and control group parents when assessed on their knowledge about their adolescents’ friends, whereabouts after school and use of free time. Of the identified studies, only one failed to observe program effects on any of the parenting specific factors measured [74]. The Youth and Alcohol program saw no program effects on parental attitudes, parental monitoring or parent-child communication [74].

### Level of stakeholder engagement

The majority of the identified parent alcohol programs reported limited levels of stakeholder engagement during program design. Specifically, over half of the programs (*n* = 8, 61%) reported methods used to inform stakeholders [68, 71, 73, 74, 79, 81, 82, 84]. For example, studies reporting on the Orebro [84] and PDFY [82] programs do not mention any form of stakeholder input during program design, reporting instead that an extensive review of the literature was conducted to inform the development of the respective programs. Three programs (23%) reported methods used to consult with stakeholders [55, 68, 76]. When designing the Self-help home ATOD communication program, formative research was conducted with parents involving a self-complete questionnaire and structured small group discussions [76]. Parents provided insights into their specific needs in terms of communicating with their adolescent (e.g. what topics to talk about) and their preferences towards the nature and delivery of the program (e.g. interactive, home-based, easy to read) [76].

Additionally, two programs utilized methods to involve, working with multiple stakeholders and developing alternative parent ideas based on stakeholder input [72, 77]. Formative research was conducted with parents representing the target audience of the Family Matters program [85]. Informal discussion with parents led to the development of a pilot test which was administered to the parents. Program materials and procedures were refined based on parent feedback [85]. Throughout this process advice was solicited from a variety of experts such as a clinical psychologist, researchers in the field of adolescent alcohol use and developers of prior family based prevention programs [77]. No programs reported use of methods to collaborate or empower stakeholders during program design.

### Level of theory utilization

Of the programs identified eight (61%) reported the use of a behavior change theory. Of those that specified a theoretical framework, three used one theory [71, 76, 79], three used two theories [55, 72, 82] and two used three or more theories [74, 77]. The most frequently identified theories were social cognitive theory [72, 74, 76, 78, 82] social learning theory [74, 77, 82] and ecological frameworks [55, 71, 72]. In terms of theory utilization level, three programs applied theory [55, 71, 79]. For example, The Substance Use Prevention Promoted by Eating family meals Regularly (SUPPER) program was developed using eco-developmental theory as a guiding framework. Key theoretical constructs, namely family bonding and social interactions were clearly identified in study reporting [71]. Five programs tested theory [72, 74, 76, 77, 82]. For example, Beatty et al., [76] report that the Self-help home ATOD Communication program incorporated key elements of SCT (e.g. improving parents’ self-efficacy to discuss alcohol related topics with their adolescent). The study used and measured several key theoretical constructs in both program implementation and evaluation (e.g. self-efficacy, knowledge and outcome expectations) [76]. No programs built theory.

### Quality assessment

A quality assessment of the identified programs was conducted using the EPHPP tool (see Table 3). Of the 13 identified programs, ten were assessed as weak in the global rating, three were assessed as moderate and none were assessed as strong. Selection bias was likely in many studies. Only one third of the programs reported representative sampling methods [68, 72, 76, 78]. However, as one of these programs reported low participation levels, only two programs were assessed as strong in regards to selection bias. Only three of the included studies described how randomisation sequences were generated [68, 69, 78] and therefore these were assessed as strong. In terms of confounders, the majority of programs (*n* = 10, 77%) reported either no baseline differences between groups or studies controlled for at least 80% of relevant confounders resulting in a strong rating. The rest of the programs did not report potential confounders or account for them in analysis and were therefore assessed as weak [69, 74, 77]. In all programs, blinding for both assessors and participants was not reported. In terms of data collection methods, almost half (*n* = 6, 46%) [55, 73–75, 78, 80] of the included studies provided evidence of the validity and reliability of the reported outcomes measures and were therefore assessed as strong. The remaining programs were assessed as either moderate (*n* = 3) [69, 76, 79] for reporting validity only or weak (*n* = 4) [68, 71, 72, 77] as they did not report validity. Regarding the retention rates of parents, only two programs were assessed as strong with more than 80% of parents completing the program.

**Table 3:**
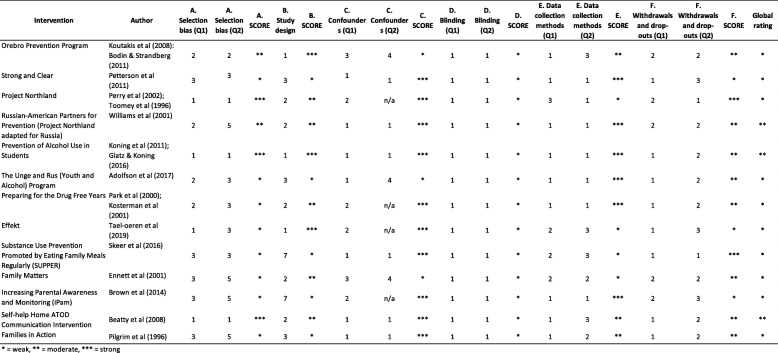
Quality assessment of included programs

## Discussion

The aims of this systematic review were threefold. First, this study aimed to examine the efficacy of parent alcohol programs on parent outcome measures. Second, this study sought to assess the level of stakeholder engagement in the design of parent alcohol programs, and finally this study aimed to assess the extent of theory utilization in program design. The discussion will address each main contribution in turn.

### Efficacy of parent alcohol programs

Each of the identified programs focused on influencing specific parenting factors associated with preventing or reducing alcohol use among adolescents including; parents’ restrictive attitudes, parent-child communication, alcohol-specific rule setting and parental monitoring. These forms of parental involvement have been described in previous studies as important protective factors for risky drinking behaviors in adolescents [17, 86].

Parental attitudes towards underage drinking changed as a result of program participation with seven of the nine programs evaluating attitudes demonstrating significant positive results. Parents restrictive attitudes towards underage drinking acts as one of the strongest protective factor for risky drinking behaviours in adolescents [87]. A change in parental attitudes is an important step towards ultimately influencing parenting specific behaviors as influencing attitudes increases the likelihood of changing behavioral intentions [88]. Furthermore, significant program effects were reported in four of the five programs measuring parent-child communication. Frequent and open alcohol specific communication between parents and adolescents can reduce adolescents’ alcohol usage, while also increasing their perceptions of the negative consequences associated with alcohol use [89].

The results showed that parent alcohol programs are effective in increasing parents’ restrictive rules concerning underage drinking. Adolescents whose parents enforce strict alcohol specific rules are less likely to engage in risky drinking behaviors [8, 90]. Furthermore, the enforcement of strict rules can still exert a protective effect from early adolescence until early adulthood [91]. Concerningly, in each of the studies assessing parental monitoring practices no program effects were observed. Operating as one of the strongest protective factors for adolescent alcohol use [6, 13, 92], parental monitoring has been found to minimize underage alcohol use [4, 93] improve adolescents’ self-efficacy to refuse alcohol [86] and improve family closeness [6].

In line with previous systematic reviews, this study provides evidence to support the efficacy of parent alcohol programs in preventing or reducing adolescent alcohol consumption [1, 13, 14] by focusing on parent specific outcome measures. However, improvements in study quality would extend confidence in reported findings. Extensive literature lends support to the notion that parenting specific behaviors play an important role in influencing and predicting adolescents’ drinking behaviors [17, 86, 93]. The results highlight emerging evidence that parent alcohol programs can achieve dual aims, providing a protective effect on adolescents in addition to changing parenting practices and attitudes. Further investigation is needed before definitive conclusions can be drawn giving heterogeneity in outcome variables and the lack of strong quality studies.

### Limited stakeholder engagement in program design

Representation and involvement of a broad range of stakeholders in the design stage of the identified parent alcohol programs was limited. The results of this study support an earlier review which identified that programs aiming to minimize harm from alcohol lacked stakeholder engagement, with a restricted focus on the people whose behavior needed to change (e.g. problem alcohol drinkers) [28]. While the importance of collaborating with key stakeholders during program design has been evidenced [21, 31, 35], this review highlights a lack of stakeholder consideration and inclusion during program design stages. Involving stakeholders during program design not only creates a sense of ownership among participants, through increased participation and empowerment [30] but may enhance effectiveness through greater acceptance and adoption of the program [21].

Over half of the identified parent alcohol programs reported methods used to inform participants, involving limited one way communications. This limited stakeholders, giving them no voice or power to influence decisions on the programs to be implemented. Collaborations with stakeholders allow program developers to tap in to the unique perspectives and insights held by various stakeholders [26] whose interests are varied. Discrepancies between expert and user views exist in the context of parent alcohol programs [94], suggesting expert designed programs may be failing to meet the unique needs of parents. Future program efforts should seek to include multiple stakeholder perspectives beyond the end user group to identify and acknowledge multiple views and resolve possible conflicts and discrepancies. For example, to assess needs and guide program development Project Northland developers consulted experts, conducted focus groups with parents and interviewed community leaders including mayors, police chiefs, school principals and local council members [95]. This allowed program developers to gain a comprehensive understanding of community expectations and views on adolescent alcohol consumption.

Stakeholder empowerment can improve innovation [36] reducing resistance towards desired change [37]. Therefore, program design processes need to evolve in order to achieve higher levels of empowerment and advance the sustainable development of programs. Thus, it is suggested that researchers consider novel methods that allow stakeholders to actively contribute during the design process as opposed to being passive participants. For example, empowering stakeholders through active collaborations such as co-design methods that provide stakeholder with the tools and a voice to design behaviour change programs of value to them [94].

### Limited theory use in parental programs

Theories are used in the development of programs to effect better outcome change [38], through influencing constructs that are known to cause specific behavior [39]. Reporting of theory use in current parent alcohol programs is lacking with only eight (61%) of the 13 programs explicitly mentioning and utilizing theory. These findings are consistent with other reviews that indicate a lack of theory use in behavior change programs [47, 96]. Theories are commonly used for audience research and segmentation [97], program development [98], message formation, promotion [98] and evaluation [46, 99]. During the formative research phase theories may be useful to assist in setting explicit aims and objectives, segmenting target audiences [100], identifying messages that resonate with the target audience and identifying important barriers and benefits to focus on [101].

Social cognitive theory is one promising theory for use in parent alcohol programs. Bandura [102] highlights the importance of SCT in behaviour change programs, emphasizing the usefulness of the theory’s constructs in influencing behaviour change. From this theoretical perspective behaviour in influenced environmental and personal factors as well as behaviour [42]. As one of the most commonly used theory’s in health behaviour research [103], it has been suggested that alcohol education programs may benefit from the application of SCT [104]. For example, the Self-help ATOD Communication program was developed based on SCT and successfully improved parent-child communication regarding alcohol and other drugs [76]. Program materials focused on key constructs of the theory such as improving parent self-efficacy to communicate to the adolescents about alcohol and increasing their knowledge of the risks associated with underage drinking [76]. However, while SCT provides health promotion researchers with one possible theoretical lens through which to examine parent alcohol program, the current findings highlight the need for more thorough application, testing and reporting of theories in behavior change programs.

### Methodological quality of included studies

Using the EPHPP quality assessment tool the methodological quality of the included programs were assessed. Ten programs were rated as weak and three as moderate. None were rated as strong. Overall the methodological quality of the included programs was low and conclusions should be interpreted with caution. Notable, methodological problems included selection biases and lack of assessor blinding. Only two programs selected participants representative of the population and achieved greater than 80% initial participation. In the included studies selection biases arose around practical issues related to the recruitment of parents. Parent were largely self-referred through convenience sampling methods such as mailouts or approaching parents at school pick up zones [71, 80], and control groups often consisted of parents who elected not to participate in program delivery [73, 79]. These findings highlight the need for increased resources dedicated to the evaluation of parent alcohol programs that permit for large scale systematic recruitment procedures.

Finally, in the current review no programs reported blinding of participants or assessors. Prior criticism has pointed to the lack of external evaluations in school based alcohol prevention programs [105]. While double blinding may not be feasible in parent based alcohol programs due to the nature of the trials, more external evaluations of parent alcohol programs are needed. Parent based alcohol program evaluations are largely evaluated by program developers who may have a vested interest in program success, thereby influencing the interpretation and reporting of results [105].

To enhance empirical evidence, future research should aim to address these issues and improve the methodological quality of parent alcohol programs. Due to the inconsistent evaluation methods and outcomes measures it was not possible to directly compare programs and make meaningful comparison of program components. Therefore, to move beyond a narrative description of programs and provide evidence towards the effectiveness of program components, more systematic reporting and evaluations of parents programs are needed. In doing so, researchers and practitioners will be provided will a more comprehensive picture of what does and does not make a program successful.

## Limitations and future research directions

The present review has several key limitations. Firstly, the study is limited by the search parameters utilized. For example, the included studies were limited to peer review journal articles, which may bias results reported. Grey literature may contribute important information and future studies may benefit from examining these sources. Second, due to the heterogeneity in the outcomes assessed, study populations, and reporting of results a meta-analysis was not possible, and a qualitative description of study outcomes was provided. Few studies included effect sizes and odds ratios, limiting our ability to compare effectiveness for parental groups. Moving forward consistent use of outcome measures is recommended. In time this would deliver consistent measures permitting meta-analytic studies to be undertaken to further enhance our understanding of program effectiveness from a parental perspective. In addition, the outcome measures relied on parent self-report data. However, self-report has been shown to be a reliable and valid method and is widely accepted in alcohol and drug prevention studies [106]. Furthermore, only 11 of39 studies received a good quality ranking and four studies had a poor quality ranking.

Workshops appear as the most common form of program delivery however often require inconvenient time commitments from parents. With the proliferation in smartphones and the creation of the ‘app economy’ [107], online and mobile based components offer an exciting opportunity for parent alcohol programs. However, only one parent alcohol program utilized online delivery methods. Smartphone applications can be utilized to deliver personalized and tailored programs to parents at a time that suits them most and with reduced time and resource requirements for program facilitators. Given well documented issues with participation and retention rates of parent in alcohol program [108], the design and delivery of mobile based parent programs offers a potential area for future research. Next, to operationalize the move towards empowering stakeholders, a clear understanding of how stakeholders can be actively engaged during program design is needed. Future research should seek to provide frameworks and tools for facilitating stakeholder engagement during program design including stakeholder identification, recruitment and empowerment. Finally, the Buyucek et al., [28] review considered stakeholder involvement in each stage of the social marketing process (i.e. formative research, implementation and evaluation) and this represents an opportunity to extend work undertaken in this review.

## Conclusion

This systematic review examined parent programs aiming to prevent and reduce adolescent alcohol use and found that parent alcohol programs can be effective in positively influencing parenting specific behaviours associated with underage drinking. However, given the mixed evidence base, study quality concerns and limited use of parent specific outcome measures, further evaluations are needed to extend the evidence base. Specifically, this review highlighted a lack of stakeholder engagement during program design and underutilization and reporting of behaviour change theories. Stakeholder insights are rarely sought or considered during program design. Involving multiple stakeholders during the design stage of programs can help to uncover additional insights to design more effective and sustainable programs. In addition, the inclusion of theory in program design and evaluation will further extend understanding of the mechanisms leading to change. This research has contributed to a better understanding of parent alcohol programs and may be of interest to public health professional and alcohol education program designers.

### Supplementary information

**Additional file 1.** Program summary. The table provides a summary of the 13 parent programs included in the review.

## Data Availability

All data generated or analysed during this study are included in this published article [and its supplementary information files].

## Appendix

**Table 4:** 42 studies included in the analysis of thirteen parent alcohol programs

No.	Program	Articles included
1	Koutakis et al., 2008 [69]; Bodin & Strandberg, 2011 [70]	Angus K, Cairns G, Eadie D, Gordon R, MacDonald L. Evaluated interventions to reduce alcohol-related harm among young people. European Commission DG SANCO; 2010 Özdemİr M, Stattin H. Does the orebro prevention programme prevent youth drinking? Addiction. 2010;107:1705–1706. Strandberg A. Evaluation of a Swedish parental prevention program: youth drunkenness, alcohol-specific parenting and gender differences. Department of Public Health Sciences; 2014 Strandberg AK, Bodin MC. Alcohol-specific parenting within a cluster-randomized effectiveness trial of a Swedish primary prevention program. Health Education. 2011;111:92–102.
2	Pettersson et al., 2011 [73]	Pettersson C, Lindén-Boström M, Eriksson C. Reasons for non-participation in a parental program concerning underage drinking: a mixed-method study. BMC Public Health. 2009;9:478–496. Pettersson C. Parents’ possibility to prevent underage drinking: studies of parents, a parental support program, and adolescents in the context of a national program to support NGOs (Doctoral Dissertation, Örebro University); 2010.
3	Perry et al., 2002 [72]; Toomey et al. 1996 [83]	Komro KA, Perry CL, Williams CL, Stigler MH, Farbakhsh K, Veblen-Mortenson S. How did project northland reduce alcohol use among young adolescents? Analysis of mediating variables. Health Education Research. 2001;16:59–70. Perry CL, Williams CL, Forster JL, Wolfson M, Wagenaar AC, Finnegan JR, McGovern PG, Veblen-Mortenson S, Komro KA, Anstine PS. Background, conceptualization and design of a community-wide research program on adolescent alcohol use: project northland. Health Education Research. 1993;8:125–136. Perry CL, Williams CL, Komro KA, Veblen-Mortenson S, Forster JL, Bernstein-Lachter R, Pratt LK, Dudovitz B, Munson KA, Farbakhsh K, Finnegan J. Project northland high school interventions: community action to reduce adolescent alcohol use. Health Education and Behavior. 2000;27:29–49. Perry CL, Williams CL, Veblen-Mortenson S, Toomey TL, Komro KA, Anstine PS, McGovern PG, Finnegan JR, Forster JL, Wagenaar AC, Wolfson M. Project northland: outcomes of a communitywide alcohol use prevention program during early adolescence. American Journal of Public Health. 1996;86:956–965. Toomey TL, Williams CL, Perry CL, Murray DM, Dudovitz B, Veblen-Mortenson S. An alcohol primary prevention program for parents of 7th graders: the amazing alternatives! Home program. Journal of Child and Adolescent Substance Abuse. 1997;5:35–54. Williams CL, Perry CL. Lessons from project northland: Preventing alcohol problems during adolescence. Alcohol Research.1998; 22:107–116. Williams CL, Perry CL, Dudovitz B, Veblen-Mortenson S, Anstine PS, Komro KA, Toomey TL. A home-based prevention program for sixth-grade alcohol use: results from project northland. Journal of Primary Prevention. 1995;16:125–147. Williams CL, Perry CL, Farbakhsh KI, Veblen-Mortenson SA. Project Northland: comprehensive alcohol use prevention for young adolescents, their parents, schools, peers and communities. Journal of Studies on Alcohol. 1999;13:112–124.
4	Williams et al., 2001 [55]	Williams Cl, Grechanaia T, Romanova O, Komro Ka, Perry Cl, Farbakhsh K. Russian-American partners for prevention: adaptation of a school-based parent-child programme for alcohol use prevention. The European Journal of Public Health. 2001;11:314–321.
5	Koning et al., 2011 [78]; Glatz and Koning, 2016 [81]	Koning IM, Maric M, MacKinnon D, Vollebergh WA. Effects of a combined parent–student alcohol prevention program on intermediate factors and adolescents’ drinking behavior: a sequential mediation model. Journal of Consulting and Clinical Psychology. 2015;83:719–727. Koning IM, van den Eijnden RJ, Engels RC, Verdurmen JE, Vollebergh WA. Why target early adolescents and parents in alcohol prevention? The mediating effects of self-control, rules and attitudes about alcohol use. Addiction. 2011;106:538–546. Koning IM, van den Eijnden RJ, Verdurmen JE, Engels RC, Vollebergh WA. A cluster randomized trial on the effects of a parent and student intervention on alcohol use in adolescents 4 years after baseline; no evidence of catching-up behavior. Addictive Behaviors. 2013;38:2032–2039. Koning IM, Vollebergh WA, Smit F, Verdurmen JE, Van Den Eijnden RJ, Ter Bogt TF, Stattin H, Engels RC. Preventing heavy alcohol use in adolescents (PAS): cluster randomized trial of a parent and student intervention offered separately and simultaneously. Addiction. 2009;104:1669–1678.
6	Adolfsen et al., 2017 [74]	Strøm HK, Adolfsen F, Handegård BH, et al. Preventing alcohol use with a universal school-based intervention: results from an effectiveness study. BMC Public Health. 2015;15:337–347. Strøm HK, Adolfsen F, Martinussen M, Handegård BH, Koposov R, Natvig H. Evaluation of a school-based alcohol intervention in Norway. License. 2013; 77.
7	Park et al., 2000 [75]; Kosterman et a., 2001 [82]	Kosterman R, Hawkins JD, Spoth R, Haggerty KP, Zhu K. Effects of a preventive parent-training intervention on observed family-interactions: proximal outcomes from preparing for the drug free years. Journal of Community Psychology*.* 1997;25:337–352.
8	Tael-Oeren et al., 2019 [68]	n/a
9	Skeer et al., 2016 [71]	n/a
10	Ennett et al., 2001 [77]	Bauman KE, Foshee VA, Ennett ST, Hicks K, Pemberton M. Family matters: a family-directed program designed to prevent adolescent tobacco and alcohol use. Health Promotion. 2001;2: 81–96.
11	Brown et al., 2014 [80]	Brown P. Increasing parental awareness and monitoring: the development and evaluation of a web-based program to empower parents to reduce underage alcohol use. Doctoral Thesis. 2010
12	Beatty et al., 2008 [76]	Beatty SE, Cross DS. Investigating parental preferences regarding the development and implementation of a parent-directed drug-related educational intervention: an exploratory study. Drug and alcohol review. 2006: 25:333–342.
13	Pilgrim et al., 1998 [79]	n/a

## Abbreviations

EPHPP: Effective Public Health Practice Project (Quality assessment tool)
PAS: Prevention of Alcohol use in Students
PDFY: Preparing for the Drug Free Years
PRISMA: Preferred Reporting Items for Systematic Reviews and Meta-Analyses
SCT: Social cognitive theory
SUPPER: The Substance Use Prevention Promoted by Eating family meals Regularly
