# Supplementary material for: A systematic review of parent based programs to prevent or reduce alcohol consumption in adolescents
Source: BMC Public Health. 2019 Nov 4;19:1451. doi: 10.1186/s12889-019-7733-x (PMC6829962; doi:10.1186/s12889-019-7733-x)
Supplement: Supplementary file 1 — Additional file 1. Program summary. The table provides a summary of the 13 parent programs included in the review. [file 12889_2019_7733_MOESM1_ESM.pdf]

Additional file 1: Program summary

Program	Trial	Substance/s	Sample	Level of stakeholder engagement	Behaviour change theory used (level of theory utilization)	Specific parenting focus of program	Intervention components directed at parents	Parent reported parenting-specific factors	Program effect on parent factors
Orebro Prevention Program	Koutakis et al., 2008	Alcohol	Sweden<br>Schools: 8<br>Parents:<br>339 intervention, 312 control<br>Parental participation:<br>Yr 7: 77%<br>Yr 8: 72%<br>Yr 9: 84%	Inform	Not reported (n/a)	Maintain and communicate strict attitudes against youth alcohol use	Intervention: Parent meetings: 5 x 30min face to face meetings with project worker (1 per semester).<br>Postal Info: 3 x mailings per semester for 5 semesters.<br>Activity catalogues: Catalogue sent to parent via post.<br><br>Control: Standard health education.	Parental restrictive attitudes towards youth drinking	Parental restrictive attitudes (baseline to 36-months) INT > CO***
Orebro Prevention Program	Bodin & Strandberg, 2011	Alcohol	Sweden<br>Schools:<br>20 intervention; 20 control<br>Parents:<br>632 intervention, 682 control<br>Parental participation: NR	Inform	Not reported (n/a)	Maintain and communicate strict attitudes against youth alcohol use	Intervention: 6 x 20min power point presentation administered by trained program presenters. Summary of each presentation mailed to parents.<br><br>Control: Standard health education	Parental restrictive attitudes towards youth drinking	Parental restrictive attitudes (baseline to 12-months and 30-months) INT > CO**
Strong and Clear	Pettersson et al., 2011	Alcohol	Sweden<br>Schools: 6<br>Parents:<br>229 intervention, 270 control<br>Parental participation: 45%<br>**participation defined as completing at least one program activity in both school yr 8 and 9*	Inform	Not reported (n/a)	Maintain parents' restrictive attitudes to youth drinking	Intervention: Parent meetings: 1 x meeting with other parents per year for 3 years.<br>Family dialogue: 2 x booklets sent via the post in first year, 1 x booklet sent per year for remaining 2 years.<br>Family meetings: 1 x meeting per year for 3 years.<br>Friend meetings: 1 x meeting per year for 3 years.	Parents restrictive attitudes towards youth drinking, parents' behaviour regarding youth drinking	Parents restrictive attitudes towards underage drinking (baseline to 27-months) INT > CO** (ES=0.32)<br>Parents behaviour regarding youth drinking (baseline to 27-months) NS

Program	Trial	Substance/s	Sample	Level of stakeholder engagement	Behaviour change theory used (level of theory utilization)	Specific parenting focus of program	Intervention components directed at parents	Parent reported parenting-specific factors	Program effect on parent factors
							Control: Standard health education		
Project Northland	Perry et al., 2002<br><br>Toomey et al., 1996	Alcohol, other drugs	USA<br>Schools: 24 school districts<br>Parents: 1793<br>Parental participation:<br>Yr 6: 90%<br>Yr 7: 33%<br>Yr 12: 20%	Involve	Ecological framework, social cognitive theory<br>(tested)	Promote parent-child communication, improve knowledge and setting up family guidelines, improve parents monitoring and supervision skills.	Intervention: Phase 1 (school year 6-8):<br>Slick Tracy Family fun night: Student's posters and projects from the program displayed<br>Amazing alternatives Home Program: booklets mailed to parents for parent and student to complete.<br>Print media campaign: local news.<br><br>Phase 2 (school year 11-12) Northland Notes for Parents: 11 x parent postcards.<br>Sound OFF! Campaign: 3 x mailouts with discussion questions regarding teen drinking<br>Print media campaign: Don't Provide! campaign and celebration posters.<br><br>Control: Phase 1 curricula in 1994-1997.	Perry et al., 2002<br>Acceptability of underage drinking, permissive norms, opposition to alcohol control policies, limited parental monitoring<br><br>Toomey et al., 1996<br>Alcohol related knowledge and attitudes, perceptions of alcohol related problems in the community, parent child communication (discussions about consequences if caught drinking, alcohol advertising, alcohol related situations, encouraging child and friends to gather at home), family rules against alcohol use, parental monitoring	Perry et al., 2002<br>Acceptability of underage drinking by guardians (at 24 months), NS<br>Permissive norms (at 24-months) INT < CO*<br>Opposition to alcohol control policies (at 24 months), NS<br>Limited parental monitoring (at 24-months), NS<br><br>Toomey et al., 1996<br>Discussions about alcohol advertising**, alcohol related situations** and consequences if caught drinking*, encouraging child and friends to gather at home*<br>(baseline to immediately post intervention) INT > CO<br>All other outcomes NS
Russian-American Partners for Prevention (Project Northland adaption)	Williams et al., 2001	Alcohol	Russia<br>Schools: 20<br>Parents: 544 intervention, 534 control<br>Parental participation: NR	Consult	Ecological framework, social cognitive theory<br>(applied)	Encourage parents to communicate and enforce strict rules about underage drinking	Intervention: 4 x take home booklets to complete with student, 1 x 1-hour family fun day poster fair<br><br>Control: Receive curriculum upon completion of initial intervention	Parents perceptions of adolescent problem behaviours, adolescents' access to alcohol, other issues (not specified)	Likely to perceive that adolescents had easier access to alcohol in their communities (baseline to 6-months) INT > CO*<br>Parents perceptions of adolescent behaviours (baseline to 6-months) NS<br>Other issues (not specified) (baseline to 6-months) NS

Program	Trial	Substance/s	Sample	Level of stakeholder engagement	Behaviour change theory used (level of theory utilization)	Specific parenting focus of program	Intervention components directed at parents	Parent reported parenting-specific factors	Program effect on parent factors
Prevention of alcohol use in students (PAS Program)	Koning et al., 2011<br><br>Glatz and Koning, 2016	Alcohol	Netherlands<br>Schools: 19<br><br>Koning et al., 2011<br>Parents: 689 parent only intervention<br>Parental participation: NR<br><br>Glatz and Koning, 2016<br>Parents: 607 parent only intervention<br>Parental participation: NR	Inform	Not reported (n/a)	Encourage restrictive parental rule-setting concerning their children's alcohol consumption	Intervention: 1 x 20min presentation given by an expert (20mins)<br>1 x meeting with parents and mentor (trained prevention professionals) to discuss rules and reach a consensus of rules<br>Summary of 1 & 2 sent to parent's home, including those not present at parent intervention<br><br>Combined intervention: Both student and parent intervention<br><br>Control: Standard health education	Rules about alcohol use, attitudes about alcohol use	Koning et al., 2011<br>Restrictive rules (baseline to 10-months) INT & PY INT > CO**<br>Strict attitudes (baseline to 10-months) INT & PY INT > CO**<br>Glatz and Koning, 2016<br>Strict rule setting about alcohol (baseline to 34-months) INT & PY INT > CO**<br>Parents confidence in their ability to prevent their adolescent drinking (baseline to 34-months) PY INT >CO**<br>Parents confidence in their ability to prevent their adolescent drinking (baseline to 34-months) INT & Y INT NS
The Unge and Rus (Youth and Alcohol) program	Adolfsen et al., 2017	Alcohol, other drugs	Norway<br>Schools: 24 intervention, 17 control<br>Parents:<br>656 intervention, 510 control<br>Parental participation:<br>1 parent meeting: 48%,<br>2 parent meetings: 31%,<br>no parent meetings: 17%	Inform	Social learning theory, social cognitive theory, theory of planned behaviour (tested)	Change parents' rules and attitudes towards adolescent alcohol use, improve parent-child alcohol specific communication, set clear limits for alcohol use	Intervention: 1 x 2-hour parent group meeting to discuss attitudes and practices related to adolescents' alcohol use<br>1 x 2-hour parent and student group meeting to discuss rules and attitudes regarding alcohol.<br><br>Control: Standard health education	Parents relationship to their adolescent, parent's knowledge about their adolescent (monitoring), parent-child alcohol specific communication	Parents attitudes towards alcohol (baseline to 4, 6 and 28 months) NS<br>Talk about the dangers of alcohol (baseline to 4, 6 and 28 months) NS<br>Ease of talking about alcohol with adolescents (baseline to 4, 6 and 28 months) NS<br>Parents' relationship with their adolescents (baseline to 4, 6 and 28 months) NS<br>Knowledge about adolescents leisure time (monitoring) (baseline to 4, 6 and 28 months) NS

**Program**	**Trial**	**Substance/s**	**Sample**	**Level of stakeholder engagement**	**Behaviour change theory used (level of theory utilization)**	**Specific parenting focus of program**	**Intervention components directed at parents**	**Parent reported parenting-specific factors**	**Program effect on parent factors**
Preparing for the Drug Free Years (PDFY)	Park et al., 2000<br><br>Kosterman et al., 2001	Alcohol, other drugs	USA<br>Schools:<br>11 intervention;<br>11 control<br>Parents;<br>144 intervention;<br>151 control<br>Parental participation:<br>94% (*attended 3 or more sessions)*<br>93% (*attended at least 4 sessions)*<br>61% (*attended all 5 sessions)*	Inform	The Social Developmental Model; integrates Social Cognitive Theory and Social Learning Theory (tested)	Improve parents' skills to explicitly establish family norms for behaviour, improve communication, improve monitoring and enforcement of family norms, manage and reduce family conflict	Intervention: 5 x 2-hour sessions (1 per week for 5 consecutive weeks)<br><br>Control: Mailed four informational leaflets describing different aspects of adolescent development (e.g., physical and emotional changes, parent-child relationships, etc.), and no intervention otherwise. Received curriculum upon completion of the study	Park et al., 2000<br>Parents' norms against substance use, family management practices (the extent of parents rule setting, monitoring and punishment for misbehaviour), family conflict (family strategies for dealing with conflict and negative feelings)<br><br>Kosterman et al., 2001<br>Rewards from parent to child (extent of approval or praise for something done well, and other expressions of appreciation and caring), family management: items assessed the extent of parents rule setting, monitoring and punishment for misbehaviour, conflict (frequency of shouting, arguing, threatening, and related behaviours)	Park et al., 2000<br>Parents' norms against substance use (baseline to 2 years) INT > CO*<br>Family management practices (baseline to 2 years) NS<br>Family conflict (baseline to 2 years) NS<br>Kosterman et al., 2001<br>Mother participants (baseline to 8 weeks)<br>Rewards to child INT > CO**<br>Monitoring and reasoning NS<br>Rules for substance use INT > CO**<br>Punishment inclination INT > CO*<br>Punishment consistency NS<br>Alcohol restrictiveness INT > CO*<br>Conflict from child NS<br>Involvement toward child INT > CO*<br>Involvement from child NS / Involvement together with child NS<br>Father participants (baseline to 8 weeks)<br>Rewards to child NS<br>Monitoring and reasoning NS<br>Rules for substance use INT > CO*<br>Punishment inclination NS / Punishment consistency NS<br>Alcohol restrictiveness NS

Program	Trial	Substance/s	Sample	Level of stakeholder engagement	Behaviour change theory used (level of theory utilization)	Specific parenting focus of program	Intervention components directed at parents	Parent reported parenting-specific factors	Program effect on parent factors
Effekt (formerly known as the Örebro Prevention Program)	Tael-Oeren et al., 2019	Alcohol	Estonia<br>Schools: 34 intervention; 32 control<br>Parents: 384 intervention; 406 control<br>Parental participation: NR	Consult	Not reported (n/a)	Maintain and communicate strict attitudes against youth alcohol use	Intervention: 6 x meetings (2 per year) to increase parents' knowledge and awareness of children-related alcohol topics and parenting skills<br>2 x newsletters per year<br><br>Control: Standard health education	Parental attitudes towards adolescent alcohol use	Parents restrictive attitudes (baseline to 18 months) INT > CO**
Substance Use Prevention Promoted by Eating family meals Regularly (SUPPER)	Skeer et al., 2016	Alcohol, marijuana, other drugs	USA<br>Schools: 5<br>Parents: 29 intervention; 35 control<br>Parental participation: NR	Inform	Eco-developmental theory (applied)	Increase the number of conversations parents have with their children about the harms associated with substance use, increase a consistent time that parents spend with their children through eating meals together	Intervention: 1 x parental handbook specific to the gender of the child<br>Website: links to additional resources<br>In-person session: 1 hour session with specialist where the main point of the handbook were reviewed<br>Home based element: half hour follow up phone call with the specialist<br>2 x text messages per week for 13 weeks (reminders and tips that reinforced handbook information)<br>1 x fridge magnet with message reinforcing the importance of family meals<br><br>Control: Receive curriculum upon completion of the study	Communication about substances (alcohol, marijuana, other drugs), family meals (frequency and duration)	Communication about alcohol (baseline to 3 months) INT > CO** (ES=1.15)<br>Communication about alcohol (baseline to 6 months) INT > CO* (ES=0.81)<br>Communication about marijuana (baseline to 3 and 6 months) NS<br>Communication about other drugs (baseline to 3 and 6 months) NS<br>Frequency of family meals (baseline to 3 and 6 months) NS<br>Duration of family meals (baseline to 3 and 6 months) NS
Family Matters	Ennett et al., 2001	Alcohol, tobacco	USA<br>Parents: 549 intervention, 658 control<br>Parental	Involve	Social learning theory, social control theory, family interaction	Promote healthy parent-child relationships, improve communication,	Intervention: 4 x Booklets mailed to parents. Booklets provide information in question and answer	Parental supervision (whether the adolescent had curfews on weekend and school nights and the parent's knowledge about	Rules about use (baseline to 3 months) INT > CO**<br>Discussions about nonfamily influences on

Program	Trial	Substance/s	Sample	Level of stakeholder engagement	Behaviour change theory used (level of theory utilization)	Specific parenting focus of program	Intervention components directed at parents	Parent reported parenting-specific factors	Program effect on parent factors
			participation: 74.1% *completed all 4 units* 8.2% *completed 3 units* 5.6% *completed 2 units* 12% *completed 1 unit*		theory, health belief model, value expectancy theory, theory of reasoned action (tested)	encourage parents to set clear and firm expectations for behaviour, increase family time spent together, improve parental monitoring	format and includes 15 activities for parents to complete. Telephone discussions with health educators following each mailing<br><br>Control: no intervention	the adolescent's friends, whereabouts after school, and use of free time), supportiveness (helping the adolescent and providing encouragement), involvement (time the parent and adolescent spent together talking and having fun), communication (the extent to which parents gave an explanation when requesting the adolescent to do something), parent's expectations for negative consequences of adolescent smoking and drinking, parent's attitude toward adolescent use, parent's encouragement not to use, parental use, rules about use, monitoring of use, availability of cigarettes and alcohol in the home, discussion about non-family influences on use (peers and the media)	use (baseline to 3 months) INT > CO** All other outcomes (baselines to 3 months) NS
Increasing Parental Awareness and Monitoring (iPAM)	Brown et al., 2014	Alcohol	USA Schools: n/a Parents: 33 intervention; 34 control Parental participation: NR	Inform	Not reported (n/a)	Improve parental awareness and monitoring	Intervention: 1 x 20-30-minute web-based program comprising 3 modules (1. Audio visual interactive quiz; 2. Modelling of communication strategies using digital media; 3. Parent monitoring checklist)<br><br>Control: Receive curriculum upon completion of the study (4 weeks post intervention completion)	Parental knowledge about underage alcohol use, parental monitoring, parent communication about alcohol, parental attitude about underage alcohol use	Parental knowledge about underage alcohol use (baseline to 4 weeks) INT > CO** Parent communication about alcohol (baseline to 4 weeks) INT > CO* Parental monitoring (baseline to 4 weeks) NS Parental attitude about underage alcohol use (baseline to 4 weeks) NS

Program	Trial	Substance/s	Sample	Level of stakeholder engagement	Behaviour change theory used (level of theory utilization)	Specific parenting focus of program	Intervention components directed at parents	Parent reported parenting-specific factors	Program effect on parent factors
Self-help home ATOD communica tion intervention	Beatty et al., 2008	Alcohol, tobacco	Australia<br>Schools:<br>14 intervention,<br>6 control<br>Parents:<br>848 intervention,<br>353 control<br>Parental participation:<br>93.6% of parents reported that they had read the intervention materials	Consult	Social Cognitive Theory (tested)	Improve parent-child communication	Intervention: 5 x communication sheets (1 every 3 weeks for 15 weeks) containing information and activities designed to encourage parents to talk with their adolescent about issues related to smoking cigarettes and drinking alcohol<br><br>Control: No intervention	Parents self-efficacy, parental knowledge, outcome expectancies, parent-child communication (frequency, recency, duration and nature of parent/child tobacco- and alcohol related communication)	Alcohol outcomes (baseline to 5 weeks)<br>Ever talked out drinking alcohol INT > CO** (OR=2.8)<br>Communicated about alcohol in the last 1- 2 months INT > CO** (OR=3.1)<br>Communicated about alcohol in the last 3-4 months INT > CO* (OR=1.9)<br>Communicated about alcohol in the last 4-5 months NS<br>Duration of communication <5mins INT>CO** (OR=2.9)<br>Duration of communication 5-10mins INT > CO** (OR=2.8)<br>Duration of communication >10mins INT > CO** (OR=3.1)<br>Level of engagement (high vs. none) INT > CO** (OR=2.3)<br>Number of topics (3-4 vs. none) INT > CO** (OR=2.2)<br>Number of topics (1-2 vs. none) NS
Families in Action	Pilgrim et al., 1998	Alcohol, tobacco, other drugs	USA<br>Schools: 4<br>Parents:<br>61 intervention<br>169 control<br>Parental participation: 71% of parents	Inform	Social developmental model (applied)	Increase positive attachment to family, increase time spent enjoying family activities, increase involvement	Intervention: 6 x 2.5hour weekly sessions. Social evening twice a year for graduates and families. Quarterly newsletter reinforcing topics taught during sessions.<br>Control: no intervention	Family cohesion, shared family activities, school attachment, participation in school activities, experience with counsellors, curriculum knowledge, attitudes towards alcohol and tobacco use by minors	Short-term effects (baseline to 6 weeks)<br>Family cohesion INT > CO**<br>Restrictive attitudes INT > CO*<br>Curriculum knowledge INT > CO**

Program	Trial	Substance/s	Sample	Level of stakeholder engagement	Behaviour change theory used (level of theory utilization)	Specific parenting focus of program	Intervention components directed at parents	Parent reported parenting-specific factors	Program effect on parent factors
			attended at least 4 out of 6 sessions			with child's school, increase willingness to talk to counsellor, increase restrictive attitudes			Short-term effects (baseline to 10 weeks)<br>Family cohesion NS<br>Restrictive attitudes INT > CO*<br>Curriculum knowledge INT > CO**<br><br>Long-term effects (baseline to 12 months)<br>Participation in school activities INT > CO**<br>Involvement in family counselling INT > CO**<br>All other outcomes NS

* P < 0.05, ** P < 0.01

INT, parent intervention; CO, control group; PY INT, combined parent and youth intervention; Y INT, youth intervention; NS, no significant difference between intervention and control groups; NR, not reported
